# Increased susceptibility to complicated pneumonia among egyptian children with FokI (rs2228570), not TaqI (rs731236), vitamin D receptor gene polymorphism in association with vitamin D deficiency: a case-control study

**DOI:** 10.1186/s12887-023-04192-x

**Published:** 2023-08-09

**Authors:** Mahitab Morsy Hussein, Enas Maher Mohamed, Tarek Mostafa Kamal, Tharwat Ezzat Deraz

**Affiliations:** 1 Pediatrics Department, Faculty of Medicine, Ain Shams University, Cairo, 1156 Egypt; 2 Medical Genetics Department, Faculty of Medicine, Ain Shams University, Cairo, Egypt

**Keywords:** VDR gene, Polymorphism, Complicated pneumonia, FokI, TaqI, Vitamin D deficiency

## Abstract

**Background:**

Determining a genetic contribution to the development of complicated community-acquired pneumonia in children may help understand underlying pathogenesis. We aimed to investigate the association between two vitamin D receptor (VDR) gene polymorphisms, FokI and TaqI, and susceptibility to complicated pneumonia in Egyptian children compared to uncomplicated pneumonia. Associations with 25 hydroxy-vitamin D serum level were studied.

**Methods:**

This was a case-control study that included 320 participants divided into 2 groups: patients and controls. The patients’ group included 100 children hospitalized with complicated pneumonia and 100 with uncomplicated pneumonia. 120 age and sex-matched apparently healthy children served as controls. The VDR FokI and TaqI polymorphisms were genotyped using polymerase chain reaction-restriction fragment length polymorphism (PCR-RFLP) technique. 25 hydroxy-vitamin D level was estimated in serum using ELISA.

**Results:**

Regarding FokI, homozygous CC genotype was more common in complicated (52%) than uncomplicated pneumonia (28%) and controls (10%) (OR = 65; 95%CI (5.13-822.63), p < 0.001) and (OR = 4.3; 95%CI (0.7-27.16), p = 0.003), respectively. Children carrying C allele possessed 3 higher odds for complicated than uncomplicated pneumonia (OR = 3.08; 95%CI (1.33–7.14), p < 0.001). Heterozygous CT genotype increased susceptibility to complicated pneumonia (OR = 13.7; 95%CI (4.6–40.1), p < 0.001), not uncomplicated pneumonia (OR = 1.56; 95%CI (0.86–2.85), p = 0.145). Among complicated pneumonia, vitamin D level was lower in CC (6.92 ± 2.6ng/ml) than CT (9.55 ± 3.2 ng/ml) and TT genotype carriers (13.13 ± 3.6ng/ml) (p < 0.001). There was no significant difference between patients and controls as regards TaqI genotypes and alleles.

**Conclusion:**

In association with vitamin D deficiency, VDR gene FokI polymorphism, not TaqI, is a genetic risk factor for complicated pneumonia in Egyptian children.

**Supplementary Information:**

The online version contains supplementary material available at 10.1186/s12887-023-04192-x.

## Background

Community-acquired pneumonia (CAP) is a global health concern accounting for high morbidity and mortality in children, mainly in developing countries [1]. Complicated pneumonia (CP) refers to infections involving lung parenchyma complicated by Parapneumonic effusion (PPE), empyema (EMP), necrotizing pneumonia (NP), lung abscess, pneumothorax, and bronchopleural fistula. PPE and EMP represent different stages of pleural inflammation. NP and lung abscess result from lung tissue liquefaction [2]. Early detection of complications is crucial. They should be suspected if there is no response to antibiotics within 3 days [3]. Despite advances in healthcare and vaccinations, frequency of CP is increasing [4]. Environmental factors were discussed for progression to complications. However, explanations are uncertain. Ethnic differences in rates of CP were found [5], implying a genetic contribution.

Early previous reports highlighted a link between vitamin D and CAP [6–8]. Vitamin D is a known growth factor for alveolar type 2 cells modulating lung tissue growth and repair [9]. Also, it exerts anti-microbial and anti-inflammatory effects through activation of nuclear vitamin D receptors (VDRs) expressed in respiratory epithelium [10]. This modifies lung inflammatory responses while enhancing defense mechanisms against respiratory pathogens [11]. Since VDR system (VDR and its ligand 1,25(OH)2 vitamin D) is a key mediator of immunity [12], genetic polymorphisms affecting VDR protein activity and expression level can influence disease susceptibility and severity.

Vitamin D receptor (VDR) is encoded by a gene with the same name located on chromosome 12 [13]. Various VDR gene polymorphisms were identified, but ApaI (rs7975232), FokI (rs2228570), TaqI (rs731236), and BsmI (rs1544410) were extensively studied [14]. FokI is a non-synonymous mutation in exon 2 of the VDR gene. The change of thymine (T) to cytosine (C), C > T single nucleotide substitution, also referred to as F > f, in the start codon sequence was found to affect structure and function of the encoded protein. It is an independent genetic marker as it is not in linkage disequilibrium with other polymorphisms. TaqI single nucleotide polymorphism (SNP), in exon 9, C > T allele change, also called T > t can influence mRNA stability [15]. Polymorphisms described in VDR gene can modulate calcidiol level and calcitriol function on binding to VDR. They can also modify response to vitamin D supplementation in health and disease [16]. Recently, a link has been highlighted between VDR SNPs and pathogenesis of various inflammatory and autoimmune conditions through its modulating effect on serum vitamin D level [17]. Polymorphisms involving VDR gene were also associated with several respiratory illnesses [18–20]. Few studies investigated association between VDR gene SNPs and CAP in Indian [21], Chinese [22], and Egyptian populations [23]. However, association with complicated pneumonia is not yet elucidated.

In this work, our objective was to study association between VDR polymorphisms, namely FokI and TaqI, and susceptibility to complicated pneumonia in Egyptian children compared to those with uncomplicated CAP. Also, relation between 25-OH vitamin D level in those children and VDR polymorphisms was assessed.

## Methods

### Study design

This was a case-control study. It was carried out on 320 Egyptian participants divided into two groups: patients and controls. Patients’ group included 200 children hospitalized with CAP, recruited from the pediatric department, children’s hospital, Ain shams university, Cairo, Egypt, during the period from October 2020 to February 2021. They were subdivided into 100 patients with complicated pneumonia and 100 patients with uncomplicated pneumonia. The control group comprised 120 age and sex-matched, apparently healthy children. They had no acute or chronic respiratory problems and were never hospitalized with pneumonia.

#### Informed consent

was obtained from participants or their legal guardians before enrollment. This study was conducted according to international guidelines of strengthening the reporting of observational studies in epidemiology, STROBE [24]. It was performed in line with principles of the Declaration of Helsinki 1975. Approval was granted by Ethics Committee of human experimentation of Ain shams university (FMASU MD 197/2019).

Children aged 1 month to 14 years were included. Pneumonia diagnosis was applied according to British thoracic society guidelines [25]. It was defined as community-acquired if infection was acquired outside hospital. For subgrouping, radiological evidence (chest x-ray and lung ultrasound) was utilized to confirm presence of local pneumonia complications and/or uncomplicated pneumonia. Complicated pneumonia was defined if massive parapneumonic effusion, empyema, necrotizing pneumonia, lung abscess, pneumothorax, or their combinations were present.

Patients with alternative respiratory diagnoses as acute bronchiolitis and microbiological evidence of pulmonary tuberculosis, underlying co-morbidities as immunodeficiency, congenital heart disease, chronic renal, hepatic, or neurological illness, associated known or suspected genetic diseases, and those on vitamin D supplementation were excluded from study.

All patients were subjected to detailed history laying stress on demographics, immunization status, antibiotics use before admission, and thorough clinical examination. Anthropometrics, including weight for age Z score, height for age Z score, and mid-arm circumference (cm) were recorded.

Initial complete blood count (CBC), C-reactive protein (CRP) level, and results of microbiological cultures were collected from patient’s recordusing a standardized data collection form.

### Laboratory work-up

Blood samples (5ml) were collected under aseptic conditions from all participants and divided into 2 tubes: an evacuated glass tube and an EDTA-containing tube. Both were stored at -20° C till further use.

Genotyping of VDR FokI (rs2228570) and TaqI (rs731236) single nucleotide polymorphisms:

Genomic DNA was extracted from whole blood samples using QIAamp DNA Blood Mini Kit (50), Catalog no.: 51,304 (Qiagen GmbH, Germany) according to manufacturer’s instructions, and DNA concentration was determined by NanoDrop 2000c Spectrophotometer (ThermoFisher Scientific, Germany).

All patients and controls were genotyped for VDR FokI and TaqI SNP using Thermo Scientific Fast Digest FokI, Catalog no.: FD2144 and Platinum ™Taq DNA polymerase, cat no: 10,966,018 (ThermoFisher Scientific, Germany), respectively. Genotyping was conducted by polymerase chain reaction-restriction fragment length polymorphism (PCR-RFLP) method using Bio-Rad T100™ Thermal Cycler. Initial denaturation at 95°C for 2 min followed by 35 cycles of denaturation at 95°C for 15 s and extension at 72°C for 30 s. Final extension of reaction ended at 72°C. PCR products of FokI and TaqI were verified by 2% agarose gel electrophoresis. Polymorphisms were detected according to digestion pattern generated for the amplified DNA fragment using restriction enzymes. FokI SNP base-pair regions were amplified using sense primer 5′- AGC TGG CCC TGG CAC TGA CTC TGC TCT-3′ and antisense primer, 5′-ATG GAA ACA CCT TGC TTG CTT CTC CCTC-3′ and TaqI SNP base-pair regions were amplified using sense primer 5-GGG ACG ATG AGG GAT GGA CAG AGC-3, and antisense primer 5-GGAAAG GGG TTA GGT TGG ACA GGA-3.

Measurement of 25-OH vitamin D level in serum was done for all patients and controls using a commercial ELISA kit (K2110, immune-diagnostic, Dutch Company, Holland) according to the manufacturer’s instructions. Subjects were segregated according to vitamin D concentration as follows: Deficiency 0–15 ng/ml, Insufficient 15–20 ng/ml and Sufficient 20–100 ng/ml [26].

### Radiological investigations

Upon enrollment, chest X-ray (CXR) and percutaneous lung ultrasound (LUS) using a GE ultrasound system (LOGIQ P9) and multifrequency linear/convex probe (7-12 MHz) were performed for all patients. Uncomplicated pneumonia was determined in CXR as dense homogenous opacity with air bronchogram involving whole or part of a lobe (lobar pneumonia) or patchy in-homogenous densities in one or both lung fields (bronchopneumonia) and as poorly circumscribed subpleural hypoechoic area of consolidation ± air bronchogram in LUS. Pleural effusion, seen in CXR as homogenous opacity involving costophrenic angle, was confirmed using LUS as anechoic fluid in the pleural cavity with or without debris. After ultrasound-guided thoracocentesis, empyema was defined as presence of pus or bacteria in the pleural fluid. PPE was defined by pleural fluid not fitting criteria of empyema. Necrotizing pneumonia was determined by presence of multiple gas-filled cavities within a pulmonary consolidation on CXR or LUS. Lung abscess was defined as a well-defined thick-walled cavity with air-fluid level; both were confirmed by computed tomography (CT) and radiologist reports.

### Statistical methods

Based on assumed prevalence of gene polymorphisms according to Roth et al. [29] and at significance level of 0.05, sample size was calculated using power analysis software (PASS II) achieving 81% power to detect expected difference in gene polymorphisms between cases and controls. Data were analyzed using Statistical Package for Social Science, version 23.0 (SPSS Inc., Chicago, Illinois, USA). Qualitative variables were expressed as number (n) and percentages (%). Quantitative data were presented as mean ± standard deviation (SD) (parametric) and Median + interquartile range (IQR) (non-parametric). Tests used for continuous variables: t-test (parametric), Mann Whitney (non-parametric) for comparison between two groups, A one-way analysis of variance (ANOVA) when comparing more than two independent groups (parametric), Kruskal Wallis test for multiple-group comparisons (non-parametric), Chi-square test and/or Fisher exact test instead of Chi-square test when expected count in any cell was less than 5 were used for comparison between categorical variables. Allele frequencies were calculated using gene counting method. Chi-square test was used to test differences between groups for each SNP genotypes and allele and to assess deviation from Hardy–Weinberg equilibrium. Odds ratios (OR) were calculated with a 95% confidence interval (CI). Multivariate binary logistic regression analysis: genotype odds ratio (OR) with 95% confidence interval was applied to assess overall association between possible risk factors and outcomes. Confidence interval was set at 95% and margin of error accepted was set at 5%,so P-value < 0.05 was considered significant.

## Results

All patients and controls enrolled in the study were well-matched regarding age, sex, and area of residence (Table 1). Complicated pneumonia (CP) (n = 100) patients’ age ranged from 8 months to 13.5 years with mean ± SD of 90.3 ± 50.5 months [60/100 were between 1 and 5 years, 28/100 were below 1 year, and 12/100 were above 5 years]. Uncomplicated pneumonia (n = 100) patients’ age ranged from 5 months to 14 years with mean ± SD of 81.5 ± 54.2 months [48/100 were between 1 and 5 years, 32/100 were below 1 year, and 20/100 were above 5 years]. CP patients were significantly more exposed to second-hand smoke when compared to uncomplicated pneumonia patients and controls (p < 0.001). Only 4% and 20% of patients received pneumococcal (PCV13) vaccine in CP and uncomplicated pneumonia groups, respectively.

**Table 1:** Demographics of patients and controls

Variable	Patients		Controls		p- value
	**Comp. pneu**. n = 100	**Uncomp. Pneu.** n = 100	n = 120		
Sex, n (%)					
Female	24 (24%)	40 (40%)	32 (26.7%)	7.111*	0.147
Male	76 (76%)	60 (60%)	88 (73.3%)		
Age (months) ^a^	90.32 ± 50.53	81.54 ± 54.26	95.53 ± 49.39	1.621•	0.682
Age category ^a^					
<1 year	4.14 ± 1.8	5.5 ± 3.2	4.9 ± 2.38		
1.5years	36.36 ± 13.56	41.1 ± 12.63	37.29 ± 14.54	1.963•	
>5years	104 ± 14.96	110 ± 25.6	102 ± 19.08		0.361
Residency, n (%)					
Rural	76 (76%)	60 (60%)	80 (66.6%)		0.053
Urban	24 (24%)	40 (40%)	40 (33.3%)	5.896*	
Exposure to second-hand smoking, n (%)					
No	20 (20%)	68 (68%)	100 (83.3%)		< 0.001
Yes	80 (80%)	32 (32%)	20 (16.7%)	95.415*	
Previous Immunization status, n (%)					
Haemophilus Influenza vaccine	84 (84%)	88 (88%)	112 (93.3%)	4.841*	0.089
Pneumococcal vaccine (PCV13)	4 (4%)	20 (20%)	72 (60%)	88.381*	< 0.001

•: Kruskal Wallis test; *: Chi-square test

^a^ *mean ± SD.*

Comp.pneu: complicated pneumonia, Uncomp.pneu: uncomplicated pneumonia

Clinical and laboratory characteristics of patients with complicated and uncomplicated pneumonia are presented in Table 2. Massive PPE (39%) was most frequent complication, followed by empyema (28%), necrotizing pneumonia (19%),and lung abscess (14%). Streptococcus pneumoniae was most common pathogen identified in cultures in both groups (52% and 40%). Mean length of hospital stay was significantly higher in patients with complicated pneumonia (p < 0.001). Among complicated pneumonia patients, 80% required pigtail ± fibrinolytic therapy or intercostal tube insertion as part of management protocol, 16% required surgical intervention in the form of pleural decortication (8%) and lobectomy (8%), 16% were admitted to ICU due to septicemia (12%) and acute respiratory failure (ARF) (4%).

**Table 2:** Clinical, laboratory, and microbiological characteristics of complicated and uncomplicated pneumonia patients

	Complicated pneumonia		Uncomplicated pneumonia		P- value
	**n = 100**		**n = 100**		
Vital data ^a^					
RR (breath/min)		45.36 ± 6.03	34.68 ± 2.82	16.04†	< 0.001
O2 saturation (%)		88.04 ± 1.51	91.68 ± 2.44	12.68†	< 0.001
Temp (°C)		39.42 ± 0.53	38.88 ± 0.42	7.98†	< 0.001
HR (beat/min)		127.68 ± 11.00	105.72 ± 10.21	14.63†	< 0.001
Anthropometrics ^b^					
Mid-arm circumference		13.5 (9–16.83)	23.83 (15.67–27.5)	4.79•	< 0.001
Weight for age Z-score		-1 (-3–0)	0 (-2–1)	4.15•	< 0.001
Height for age Z-score		0 (-2–2)	0 (-1–1)	3.07•	< 0.001
Pre-admission antibiotics, n (%)					
No		60 (60%)	20 (20%)	31.68*	< 0.001
Yes		40 (40%)	80 (80%)		
Length of hospital stay (days) ^a^		11.48 ± 1.92	8.68 ± 1.41	11.75†	< 0.001
Range		9–15	7–11		
Type of complications, n (%)					
Massive PPE		39 (39%)	-		
Empyema		28 (28%)	-		
NP + pneumothrx ± empyema		19 (19%)	-	-	-
Lung abscess		14 (14%)	-		
Causative organism, n (%)					
Strep. pneumoniae		52 (52%)	40 (40%)	2.43*	0.119
MERSA		12 (12%)	8 (8%)	0.50*	0.480
Mycoplasma		28 (28%)	20 (20%)	1.34*	0.246
H. influenzae		8 (8%)	32 (32%)	16.53*	< 0.001
Laboratory results ^a^					
TLC (thousand/cmm)		13.24 ± 0.097	11.80 ± 1.19	12.06†	< 0.001
Hb (g/dl)		10.00 ± 1.91	11.16 ± 1.60	4.65†	< 0.001
PLT (thousand/cmm)		390.12 ± 14.77	365.60 ± 18.91	10.21†	< 0.001
CRP (mg/l)		24.92 ± 4.08	23.92 ± 4.13	1.72†	0.087
Outcome, n (%)					
Surgical intervention		16 (16%)	0 (0%)	15.28*	< 0.001
ICU admission		16 (16%)	8 (8%)	2.32*	0.128
Mortality		4 (4%)	0 (0%)	2.29*	0.130

† t-Independent Sample t-test; •Mann-Whitney test; * Chi-square test; Fisher’s exact test

*a: mean ± SD; b: median (IQR)*

*PPE: parapneumonic effusion; NP: necrotizing pneumonia; pneumothrx: pneumothorax; Strep.pneumoniae: streptococcus pneumoniae; MERSA: methicillin-resistant staphylococcus.aureus;H.influenzae: Haemophilus influenzae; TLC: total leucocytic count; Hb:haemoglobin; PLT: platelets; CRP: C-reactive protein; RR: respiratory rate; O2:oxygen; Temp: temperature; HR: heart rate; ICU: intensive care unit*

Distribution of VDR FokI and TaqI SNP genotypes and alleles in both patients ‘groups and controls are shown in Table 3. Regarding FokI genotypes distribution, a significant difference was found between both patients’ groups and controls. The dominant genotype among CP, uncomplicated pneumonia, and controls is homozygous CC (52%), heterozygous CT (40%), and TT(60%) respectively. Homozygous CC genotype is significantly common in CP (52%) and uncomplicated pneumonia (28%) compared to controls (10%) (OR = 65;(95%CI:5.13–822.6), p < 0.001 and (OR = 13.7;(95%CI:4.6–40.1), p < 0.001, respectively. C allele carriers are 3 folds more susceptible to complicated than uncomplicated pneumonia (OR = 3;(95%CI:1.33–7.14), p < 0.001. Heterozygous Ff genotype increased susceptibility to complicated pneumonia (OR = 13.7; 95%CI (4.6–40.1), p < 0.001), not uncomplicated pneumonia (OR = 1.56; 95%CI (0.86–2.85), p = 0.145). There was no significant difference between both patient groups and controls as regards TaqI genotypes and alleles distribution.

**Table 3:** Distribution of VDR FokI and TaqI genotypes, alleles, and 25-OH vitamin D serum level among complicated, uncomplicated pneumonia patients and controls

Variable	Comp. pneu.	Uncom. pneu.	Controls	Comp. vs. Uncom	Comp. vs. Controls	Uncom. vs. Controls
	**n = 100**	**n = 100**	**n = 120**	**OR (95% CI); p-value**	**OR (95% CI); p-value**	**OR (95% CI); p-value**
FokI Polymorphism Genotypes, n (%)						
TT	4 (4%)	32 (32%)	60 (50%)	**Referent**		
CT	44 (44%)	40 (40%)	48 (40%)	8.8 (0.92–83.36); p < 0.001	13.7 (4.6–40.1); p < 0.001	1.5 (0.86–2.85); p = 0.145
CC	52 (52%)	28 (28%)	12 (10%)	14.8 (1.5-144.2); p < 0.001	65 (5.13-822.63); p < 0.001	4.3 (0.70-27.16); p = 0.003
Alleles frequency, n (%)						
T	52 (26%)	104 (52%)	168 (70%)	3 (1.33–7.14); p < 0.001	**Referent** 6.6 (2.87–15.37); p < 0.001	2.1 (0.98–4.71); p < 0.001
C	148 (74%)	96 (48%)	72 (30%)			
TaqI Polymorphism Genotypes, n (%)						
tt	12 (12%)	12 (12%)	16 (13.3%)	**Referent**		
Tt	12 (12%)	12 (12%)	16 (13.3%)	1 (0.10–9.61); p = 1.000	1 (0.21–8.31); p = 1.000	1 (0.23–5.81); p = 0.733
TT	76 (76%)	76 (76%)	88 (73.3%)	1 (0.18–5.60); p = 1.000	1 (0.23–5.81); p = 0.733	1 (0.23–5.81); p = 0.733
Alleles frequency, n (%)						
t	36 (18%)	36 (18%)	48 (20%)	1 (0.36–2.77); p = 1.000	**Referent** 1.1 (0.44–2.97); p = 0.595	1.1 (0.44–2.97); p = 0.595
T	164 (82%)	164 (82%)	192 (80%)			
^a^25-OH vitamin D serum level (ng/ml)	9.01 ± 4.5	20.52 ± 8.4	52.37 ± 15.7	P < 0.001•	P < 0.001•	P < 0.001•

*•: t-Independent Sample t-test; OR: Odds ratio, CI: Confidence interval*

*a: mean ± SD*

*Comp.pneu.: complicated pneumonia; Uncom. pneu.: uncomplicated pneumonia; Comp.: complicated; Uncom.: uncomplicated; vs.: versus*

Empyema and necrotizing pneumonia were more frequent in FokI homozygous CC genotype carriers compared to CT and TT genotypes. TT genotype conferred protection against empyema and necrotizing pneumonia (p < 0.001, 0.04). Heterozygous CT genotype was associated with higher risk of massive PPE (P < 0.001). Lung abscess diagnosis was not significantly associated with FokI genotypes (p = 0.42). In CP, homozygous CC genotype carried a higher risk of ICU admission (p = 0). surgical intervention was more frequent in patients carrying homogenous homozygous CC genotype than heterozygous CT and TT genotype (p < 0.001). Mortality occurred in patients with homozygous CC genotype (8%), however, was insignificantly associated with FokI genotypes (p = 0.14) (Table 4).

**Table 4:** Association of VDR FokI polymorphism genotypes with complications and outcome in complicated pneumonia patients

	VDR FokI genotypes			^*^	p-value
	CC (n = 52)	CT (n = 44)	TT (n = 4)		
Complications, n (%)					
Massive PPE	10 (19.2%)	35 (79.5%)	3 (75%)	35.943	< 0.001
Empyema	30 (57.7%)	7 (15.9%)	1 (25%)	17.960	< 0.001
NP + pneumothrx ± EMP	9 (17.3%)	1 (2.3%)	0 (0%)	6.449	0.040
Lung abscess	4 (7.7%)	1 (2.3%)	0 (0%)	1.693	0.429
Outcome, n (%)					
Surgical intervention	46 (88.5%)	10 (22.7%)	1 (25.0%)	43.758	< 0.001
ICU admission	20 (38.5%)	5 (11.4%)	0 (0%)	10.723	0.005
Mortality	4 (7.7%)	0 (0%)	0 (0%)	3.846	0.146

^*^ *Chi-square test; Fisher’s exact test*

*PPE: parapneumonic effusion, NP: necrotizing pneumonia, pneumothrx: pneumothorax; EMP: empyema; ICU: intensive care unit, VDR: vitamin D receptor*

Table 2 shows that mean 25-OH vitamin D serum level was significantly lower in CP and uncomplicated pneumonia versus controls (p < 0.001). Vitamin D deficiency was more common among CP (60%) and uncomplicated pneumonia (40%) compared to controls (19%). Vitamin D deficient patients have about 1.7 folds higher susceptibility to develop CP than uncomplicated pneumonia (OR = 1.69;(95% CI:0.91–3.14), p = 0) (Figure S1). In CP, C allele was associated with lower mean 25-OH vitamin D level versus T allele (p < 0.001). In contrast, TaqI genotypes and alleles were insignificantly associated with vitamin D level (p = 0.13, 0.06) (Table 5). Similar findings were reported in uncomplicated pneumonia (Table S1).

**Table 5:** Association between serum 25-OH vitamin D level with VDR FokI and TaqI genotypes and alleles in complicated pneumonia group

VDR Polymorphism	25-OH vitamin D serum level (ng/ml)		P value
FokI genotypes		14.83*	< 0.001
CC	6.92 ± 2.61		
CT	9.55 ± 3.29		
TT	13.13 ± 3.68		
FokI alleles		6.20•	< 0.001
C	8.24 ± 2.95		
T	11.34 ± 3.49		
TaqI genotypes		2.07*	0.131
TT	7.40 ± 3.46		
Tt	8.67 ± 1.15		
tt	9.38 ± 5.08		
TaqI alleles			
Tt	7.98 ± 2.81	1.86•	0.064

*: One way ANOVA; •: t-Independent Sample t-test

*VDR: vitamin D receptor; 25-OH: 25 hydroxy*

Multivariate logistic regression analysis showed that C allele carriage (p = 0.035), low vitamin D level (p = 0.033), smoking exposure (p = 0.047), anemia (p = 0.036), failure of pneumococcal vaccination (p = 0.029) and delayed antibiotic administration (0.048) were independent predictors for complicated pneumonia. Furthermore, homozygous CC genotype carriers are 4.5-fold more susceptible to complicated than uncomplicated pneumonia (Table 6).

**Table 6:** Multivariate binary logistic regression analysis of risk factors affecting complicated pneumonia versus uncomplicated pneumonia

Variable	β	SE	Wald test	P-value	OR	95% C.I.	
						Lower	Upper
**Exposure to second-hand smoking**	0.284	0.199	3.176	0.047	1.934	1.271	3.160
**No Pneumococcal vaccine (PCV13)**	1.735	0.388	8.235	0.029	2.041	0.963	7.236
**Mid-arm circumference**	-1.040	0.257	0.904	0.715	1.785	0.491	3.757
**Weight for age Z-score**	-0.221	0.055	1.487	0.391	1.571	1.120	2.538
**Delayed antibiotic administration**	0.410	0.208	2.853	0.048	2.176	1.803	3.726
**Hemoglobin level (g/dl)**	0.805	0.541	4.309	0.036	2.026	1.080	6.345
**25-OH vitamin D level (ng/ml)**	1.995	0.960	9.471	0.033	2.347	1.108	8.322
**Fok1 Polymorphism** **Genotypes**							
**CT**	0.204	0.103	4.500	0.043	2.716	2.268	3.619
**CC**	2.524	1.020	9.351	0.021	4.496	3.343	7.916
**Alleles**							
**C**	0.521	0.264	3.623	0.035	2.764	2.290	4.732

*β: Regression coefficient, SE: Standard error, OR: Odds ratio, CI: Confidence interval*

## Discussion

Development of pneumonia-associated complications is a major cause of prolonged morbidity in children and increased financial burden, especially in developing countries. Identifying children with CAP at risk for progression to complications may help guide clinicians, as more aggressive therapy is required [5]. Host factors affecting immunity can be implicated in this outcome. Vitamin D is considered a key regulator of innate immune response against microbial pathogens. It exerts genomic effects through binding to nuclear VDR, controlling transcription of multiple gene products. Recently, it was found that most cells, particularly of the immune system, express VDR and 1α hydroxylase. At site of infection, VDR gene increases local VDR expression and active vitamin D production in innate immune cells, including alveolar macrophages. This enhances secretion of cathelicidin and other antimicrobial peptides [27]. Vitamin D also has known effects on B, T lymphocytes, and dendritic cells, modulating adaptive immune response and preventing uncontrolled inflammation that can potentially result in tissue damage [28]. Some VDR gene polymorphisms were described to influence disease susceptibility, course, and outcome [16, 17]. As far as we know, this study is unique in elucidating relationship between occurrence of local pneumonia complications and two VDR polymorphisms, namely FokI and TaqI. Our findings pointed out that FokI, but not TaqI, SNP in the VDR gene could be a genetic risk factor for progression to complicated pneumonia in Egyptian children. Callel can increase susceptibility to pneumonia with higher odds for pneumonia complications,it was the dominant allele among children with complicated pneumonia (74%). Homozygous CC genotype was dominant among children with complicated pneumonia (52%), its presence increased odds for complications about 15 times higher than uncomplicated pneumonia. Heterozygous CT genotype carriers possessed increased susceptibility to complicated, not uncomplicated pneumonia. Conversely, the wild-type T allele (26%) and TT genotype (4%) were reported with a significantly lower frequency in complicated pneumonia. Empyema (57.7%) and PPE (79.5%) were frequent among homozygous CC and heterozygous CT carriers, respectively. TT genotype conferred protection against Empyema (25%) and necrotizing pneumonia (0). Abouzeid et al. [23] studied VDR FokI distribution among Egyptian children with uncomplicated pneumonia, his findings were concordant with our results. However, this was against findings of Awasthi et al. [21] from India, who found that homozygous CC genotype was less frequent among CAP compared to controls, and the T allele carried a higher risk for uncomplicated pneumonia in his population. Roth et al. [29] also concluded that Canadian children carrying TT genotype had higher odds for acute lower respiratory tract infections, mainly bronchiolitis, than CC genotype. These inconsistent findings may be related to variable genotype frequencies among populations with different ethnic backgrounds and diverse genetic-environmental interactions.

FokI SNP in VDR gene results in two potential translation sites due to thymine (T) to cytosine (C) substitution at initiation codon. The allelic variants of FokI polymorphism generate 2 structurally different VDR proteins.

The presence of C allele creates a truncated protein 3 amino acids shorter (424 amino acids) instead of wild-type protein (427 amino acids) produced by T allele [30]. Studies have shown that C-short protein has a higher transcriptional activity than T-long protein [31]. This could modify effect established by vitamin D in various tissues due to increased VDR functionality. Etten et al. [32] found that FokI SNP genotypes interacted differently as regards transcriptional activity of immune-specific transcription factors, immune cell proliferation, and cytokine secretion, also suggesting a possible contribution of the VDR system to immune response outcome. Physiological role of vitamin D signaling in immune system regulation is mediated by adequate vitamin D concentration. Thus, vitamin D deficiency is a crucial factor in immune system functioning [33]. Exogenous and endogenous vitamin D is activated in the liver and kidney to produce the active form, 1,25 (OH)2 vitamin D, whose plasma level is regulated by its precursor 25-OH vitamin D and enzymatic activity. Description of vitamin D deficiency and insufficiency is still controversial. Also, the optimal concentration of 25-OH vitamin D required for proper function of immune system is not yet defined [34]. In our study, 92% and 80% of patients with complicated and uncomplicated pneumonia, respectively, had poor vitamin D status. This is in line with previous reports that confirmed the association between inadequate vitamin D levels and increased risk for CAP [35, 36]. Moreover, vitamin D deficient status was associated with an increased risk of complicated pneumonia by about 1.7 folds. Also, patients with complicated pneumonia showed a very low serum vitamin D level compared to uncomplicated pneumonia patients and controls. FokI C-variant carriers showed significantly lower vitamin D levels compared to T-variant carriers in complicated and uncomplicated pneumonia. Abouzeid et al. [23] reported the same finding. Similarly, Monticielo et al. [15] attributed this association to the differential biological activity of both alleles with higher consumption for the more active C-variant. Among complicated pneumonia patients, considering the low vitamin D levels, homozygous CC genotype carriers showed the worst prognosis in the form of more severe disease and complications occurrence requiring ICU admission (16%) and unresolved infection requiring surgical intervention (16%). This may be explained by immune dysregulation cascade coupled with vitamin D deficiency. Vitamin D exerts an inhibitory action on adaptive immune system by blocking maturation of dendritic cells, inhibiting differentiation of Th1 and Th17 T cells by suppressing IL-12, IL-23, and IL-6 production. This reduces IFNγ, IL-2, and IL-17, limiting T lymphocyte recruitment and proliferation. Decrease in IL-12 increases Th2 cells which produce IL-4, IL-5, and IL-13, shifting balance from Th1 to Th2 profile. Furthermore, it increases regulatory T cells. Such regulation is involved in immune tolerance, preventing severe disease [37].

This work showed that VDR gene TaqI SNP could not be a risk factor for pneumonia or local pneumonia complications in Egyptian children. There was no significant difference between patients and controls as regards TaqI genotypes and alleles distribution. Also, they were not associated with serum 25-OH vitamin D levels. To our knowledge, this was first Egyptian study to report these findings. Awsathi et al. [21], Roth et al. [29], and Mansy et al. [38] supported our findings in Indian, Canadian, and Saudi children, respectively.

Erlichman et al. [39] and Ooi et al. [40] reported increased frequency of pediatric complicated CAP in their localities, this is also a significant observation in our country. Ooi et al. [40] found that delayed antibiotic administration and parental smoking were risk factors for local pneumonia complications in Malaysian children. This was also a significant observation in our study. Similarly, Krenke et al. [41] and Elemraid et al. [42] linked antipyretic use to control fever and delayed outpatient antibiotics to local pneumonia complications. In our study, pneumococcal vaccine rates were significantly lower among all pneumonia patients. Streptococcus pneumoniae was significantly higher in complicated pneumonia. Similar findings were reported by Erlichman et al. [39]. Clinical parameters previously identified [3, 41] as risk factors for complicated pneumonia, mainly malnutrition, hypoxia, higher temperature, respiratory rate, and lower hemoglobin levels, were also reported in this work. The main limiting point include small sample size, single-center contribution, other VDR polymorphisms were not include, and presence of multiple confounding factors making cause-effect assumption difficultWe recommend further multicenter studies on a larger scale to confirm proposed association.

## Conclusion

The current study supported that in presence of vitamin D deficiency, C-variant of FokI polymorphism in VDR gene may represent a genetic risk factor for progression to local pneumonia complications. TaqI polymorphism alleles do not increase susceptibility to pneumonia or pneumonia complications in Egyptian children.

### Electronic supplementary material

Below is the link to the electronic supplementary material.

Supplementary Material 1

## Data Availability

All data generated or analyzed during this study are included in this published article and its supplementary information file.

## List of Abbreviations

VDR: Vitamin D receptor
SNP: Single nucleotide polymorphism
CAP: Community-acquired pneumonia
CP: Complicated pneumonia
PPE: Parapneumonic effusion
EMP: Empyema
NP: Necrotizing pneumonia
