# Supplementary material for: Increased susceptibility to complicated pneumonia among egyptian children with FokI (rs2228570), not TaqI (rs731236), vitamin D receptor gene polymorphism in association with vitamin D deficiency: a case-control study
Source: BMC Pediatr. 2023 Aug 9;23:394. doi: 10.1186/s12887-023-04192-x (PMC10410927; doi:10.1186/s12887-023-04192-x)
Supplement: Supplementary file 1 — Supplementary Material 1 [file 12887_2023_4192_MOESM1_ESM.docx]

**Additional file 1**

**Table S1 Association between serum 25-OH vitamin D level with VDR Fok1 and Taq1 genotypes and alleles in uncomplicated pneumonia group**

| VDR Polymorphism | | 25-OH vitamin D serum level (ng/ml) |  | P value |
| --- | --- | --- | --- | --- |
| FokI genotypes  CC  CT  TT |  | 13.66±9.47 | 21.61* | <0.001 |
|  |  | 21.69±6.83 |  |  |
|  |  | 25.27±3.84 |  |  |
| Fok1 alleles  C  T |  | 17.68±8.15 | 5.79• | <0.001 |
|  |  | 23.48±5.34 |  |  |
| TaqI genotypes  TT  Tt  tt |  | 19.83±9.26 | 1.25* | 0.291 |
|  |  | 22.35±2.52 |  |  |
|  |  | 23.33±3.51 |  |  |
| TaqI alleles  T  t |  | 21.79±5.89 | 1.03• | 0.300 |
|  |  | 22.84±3.02 |  |  |

**: One way ANOVA; •: t-Independent Sample t-test*

*VDR: vitamin D receptor*

**Figure S1 Vitamin D status among studied subjects**

*OR: odds ratio, CI: confidence interval*
